# A Case of Pandoraea apista Bacteremia in an Immunosuppressed Ventriculoperitoneal (VP) Shunt Patient in Dubai Hospital, UAE

**DOI:** 10.7759/cureus.99389

**Published:** 2025-12-16

**Authors:** Anju Nabi, Rubina Lone, Adnan Alatoom, Maya Habous, Bhavna Mathur, Rubina Monga, Sujth Joseph

**Affiliations:** 1 Laboratory Medicine, Dubai Hospital, Dubai Health Authority, Dubai, ARE; 2 Pathology and Microbiology, Al Jalila Children’s Hospital, Mohammed Bin Rashid University of Medicine and Health Sciences, Dubai, ARE; 3 Pathology and Microbiology, Latifa Hospital, Mohammed Bin Rashid University of Medicine and Health Sciences, Dubai, ARE; 4 Pathology, Laboratory Medicine, and Microbiology, Rashid Hospital, Mohammed Bin Rashid University of Medicine and Health Sciences, Dubai, ARE; 5 Pathology, Laboratory Medicine, and Microbiology, Latifa Hospital, Mohammed Bin Rashid University of Medicine and Health Sciences, Dubai, ARE; 6 Microbiology and Laboratory Medicine, Al Jalila Children’s Hospital, Mohammed Bin Rashid University of Medicine and Health Sciences, Dubai, ARE

**Keywords:** bacillus, bacteremia, covid, cystic fibrosis, extended-spectrum β-lactamase (esbl), maldi-tof, minimum inhibitor concentration, pandoraea apista, pneumonia

## Abstract

*Pandoraea* species are multidrug-resistant (MDR) Gram-negative bacteria increasingly recognized as opportunistic pathogens beyond cystic fibrosis (CF). To our knowledge, we report the first documented case of *Pandoraea apista* bacteremia in the Middle East, occurring in a 41-year-old non-CF woman with post-revision ventriculoperitoneal (VP) shunt hydrocephalus, metastatic cervical carcinoma, and renal failure. Presenting with fever, hematuria, and obstructive uropathy, the patient developed catheter-related bloodstream infection (CRBSI) and urinary tract infection. *P. apista* was identified via MALDI-TOF MS (matrix-assisted laser desorption/ionization time-of-flight mass spectrometry) from blood and urine cultures, displaying resistance to ceftazidime and meropenem but susceptibility to ciprofloxacin, which led to clinical improvement.

This case highlights the emerging threat of *P. apista* in immunocompromised, non-CF patients with indwelling devices, emphasizing the critical role of precise microbiological identification and susceptibility-guided therapy in managing such infections.

## Introduction

*Pandoraea apista* is a Gram-negative, catalase-positive, aerobic, motile bacillus with a single polar flagellum and is classified within the genus *Pandoraea* of the family *Burkholderiaceae*. Members of this genus are non-spore-forming, non-nitrate-reducing, and non-lactose-fermenting rods that display notable metabolic versatility, growing optimally at 30-37°C on standard laboratory media. They exhibit characteristic biochemical activities - including leucine arylamidase, catalase, and acid and alkaline phosphatase - which support their adaptability across diverse ecological and clinical environments.

Initially recognized as colonizers and pathogens in individuals with cystic fibrosis (CF), *Pandoraea* species have increasingly emerged as clinically significant multidrug-resistant (MDR) organisms in non-CF populations as well [1]. Their intrinsic resistance to multiple antimicrobial classes has been well documented, often complicating therapeutic decision-making and contributing to adverse clinical outcomes [2]. Severe infections, including bacteremia and lower respiratory tract infections, have been reported in both CF and non-CF patients with complex comorbidities and prolonged hospitalization [3]. Cases of *P. apista* bacteremia, including rare reports in non-CF patients, further highlight its pathogenic potential [4]. A notable virulence factor of *Pandoraea* spp. is their capacity to form robust biofilms, particularly on indwelling medical devices such as central lines and catheters, which may necessitate device removal to achieve infection resolution [5].

In this report, to our knowledge, we present the first documented case of *P. apista* bacteremia in the Middle East, occurring in a non-CF patient with significant comorbidities. This case highlights the emerging clinical relevance of this rare pathogen and underscores the ongoing challenges in its detection, antimicrobial management, and infection control.

## Case presentation

A 41-year-old female was transferred from Ras Al Khaimah Hospital to Dubai Hospital in June 2025 with a three-day history of abdominal pain, dizziness, and vaginal bleeding. Her medical history was significant for hypertension, stage 4 chronic kidney disease (CKD), metastatic cervical carcinoma treated with chemotherapy and brachytherapy, and hydrocephalus managed with a ventriculoperitoneal (VP) shunt, which was revised two months prior to admission. She had undergone a total hysterectomy one month earlier for cervical carcinoma. On admission, she presented with fever, with a temperature of 38.2°C, frank hematuria, clot retention, and severe anemia. A Foley catheter was in place with ongoing cystoclysis for suspected obstructive uropathy. Neurological examination revealed drowsiness but preserved orientation (Glasgow Coma Scale 14/15), attributed to uremic encephalopathy secondary to obstructive uropathy. Acute renal failure was confirmed, and hemodialysis was initiated via a right internal jugular vein (IJV) catheter on day 2 of admission.

On clinical examination, the vital signs showed a temperature of 36.6°C (after antipyretics), a heart rate of 99 BPM, a respiratory rate of 18/min, a BP of 110/63 mmHg, and an oxygen saturation of 99%.

On general appearance, she was pale and drowsy but oriented to time, place, and person. The abdominal examination showed a soft, non-tender abdomen with no organomegaly and a Foley catheter in situ. The neurological examination showed that the patient was drowsy with no focal deficits, and the VP shunt was palpable without signs of local infection. There was no respiratory distress, and the cardiovascular system was stable (Table 1).

**Table 1:** Laboratory findings

Test	Result	Reference Range	Interpretation
Hemoglobin	7.9 g/dL	12-15 g/dL	Low
White blood cell count	19.3 × 10³/μL	4-11 × 10³/μL	High
Platelets	180 × 10³/μL	150-400 × 10³/μL	Normal
C-reactive protein (CRP)	87.5 mg/L	<5 mg/L	High
Procalcitonin	6.73 μg/L	<0.5 μg/L	High
Creatinine	2.8 mg/dL	0.6-1.1 mg/dL	High
Blood urea nitrogen (BUN)	45 mg/dL	7-20 mg/dL	High
Phosphorus	1.76 mmol/L	0.81-1.45 mmol/L	High
Iron	6.7 μmol/L	10-30 μmol/L	Low
Ferritin	150 ng/mL	15-150 ng/mL	High-normal

**Figure 1:**
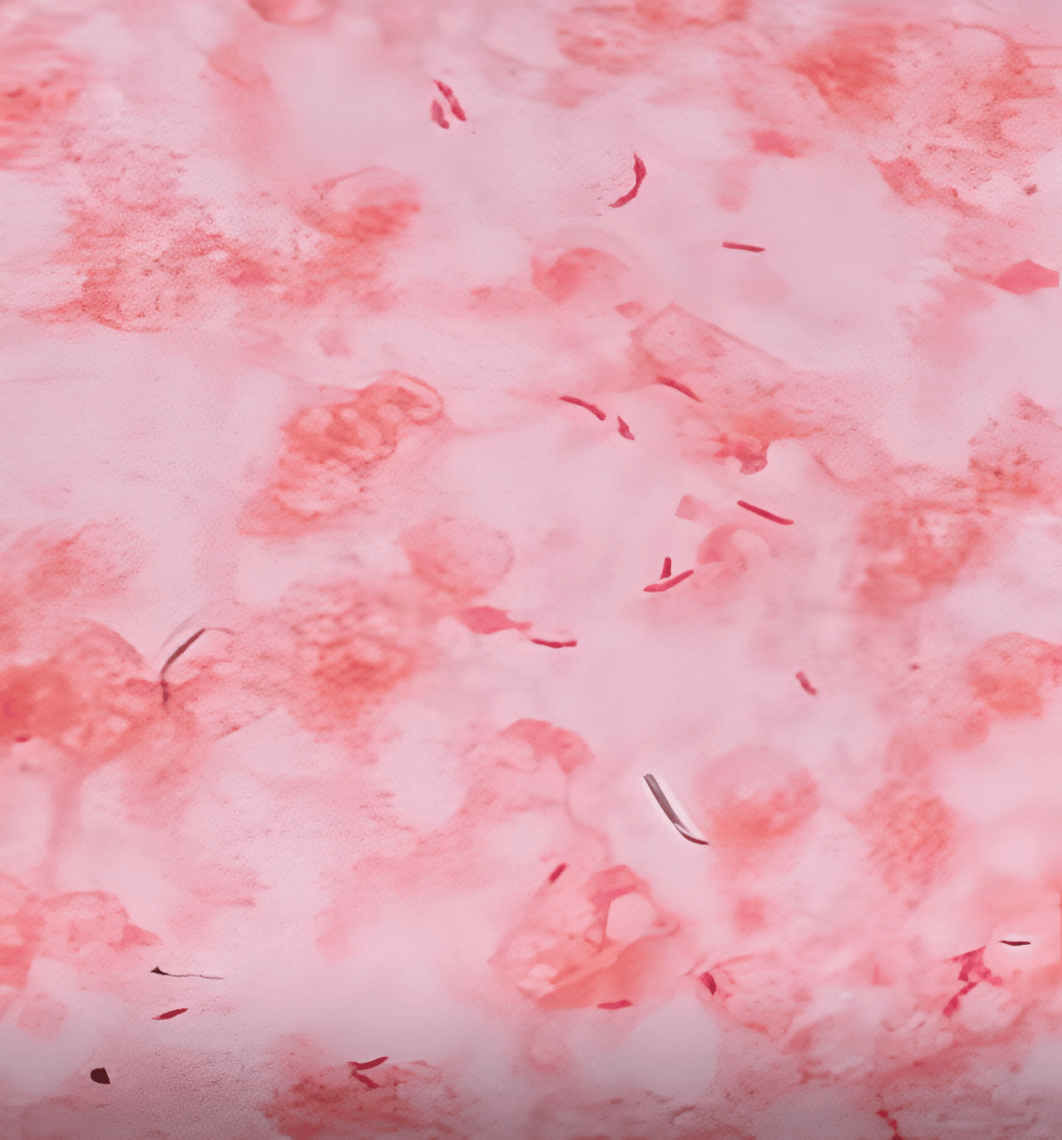
Gram stain of colonies grown on culture plates from urine and blood samples

Microbiological urinalysis showed significant pyuria (30-35 WBC/HPF) and positive leukocyte esterase, with gross hematuria. Gram stain of the urine and blood culture colonies showed Gram-negative rods, as shown in Figure 1.

**Figure 2:**
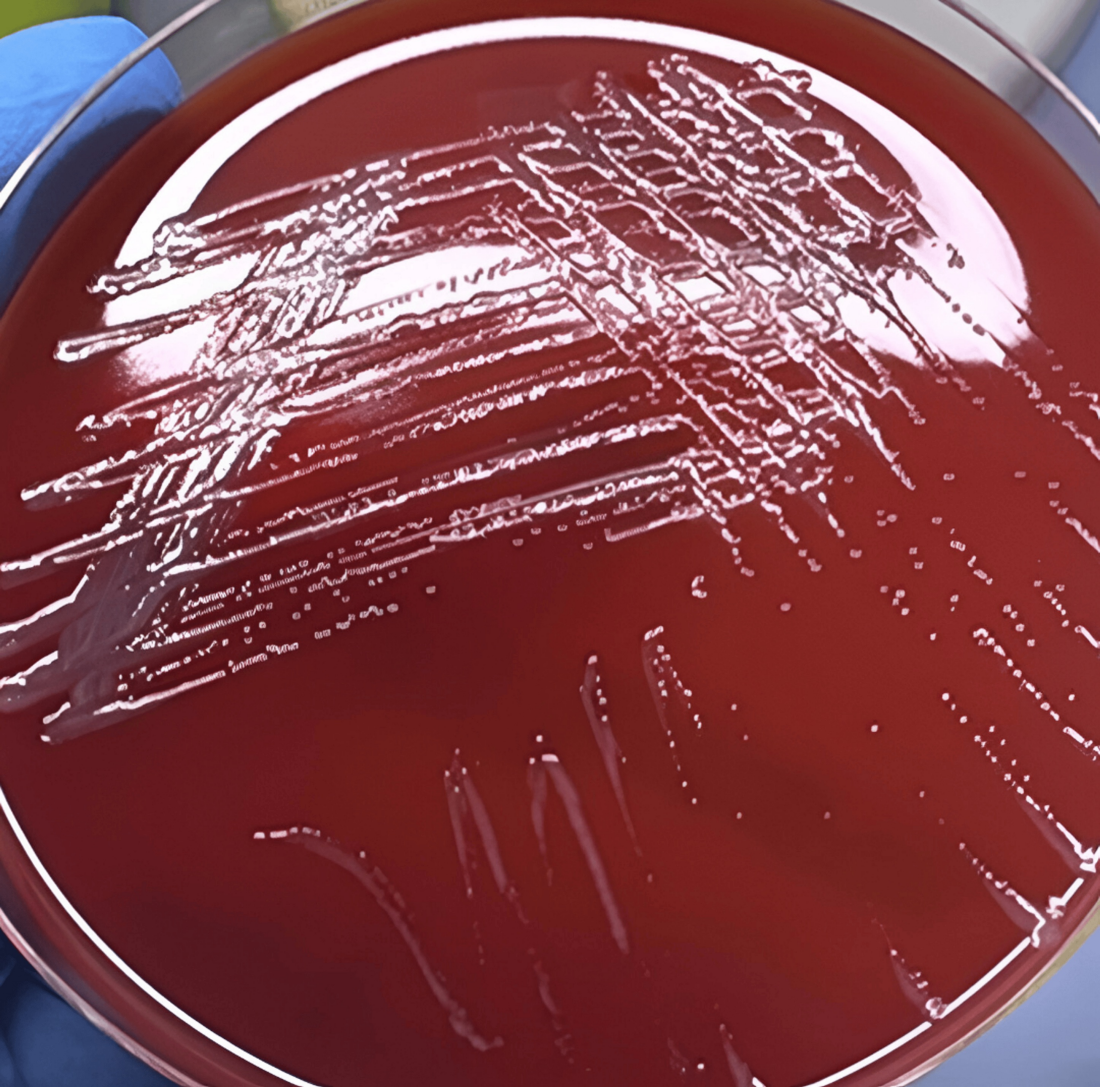
Sheep blood agar showing non-hemolytic, small, off-white, smooth, moist colonies

Nephrostomy urine processed for culture and two sets of blood cultures grew Gram-negative rods, which were identified as *P. apista* (99.9% probability) by MALDI-TOF MS (matrix-assisted laser desorption/ionization time-of-flight mass spectrometry). After 22 hours at 35°C, the colony characteristics on sheep blood agar showed non-hemolytic, small (1-2 mm), off-white, smooth, moist colonies (Figure 2).

**Figure 3:**
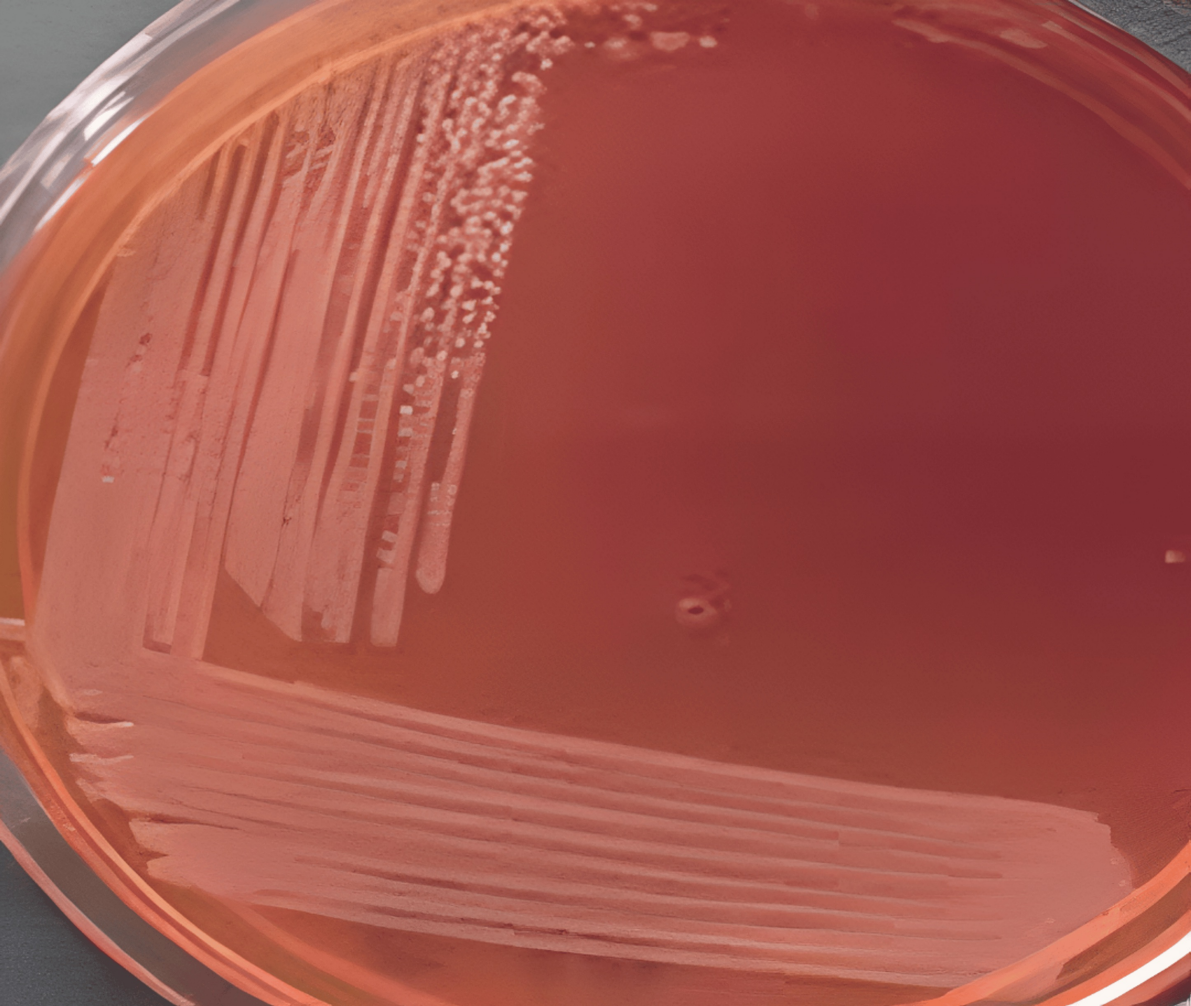
MacConkey agar showing small, round, well-defined, non-lactose-fermenting colonies

On MacConkey agar, small, round, well-defined, non-lactose-fermenting colonies were seen, as shown in Figure 3.

Antibiotic sensitivity testing, performed according to Clinical and Laboratory Standards Institute (CLSI) guidelines using VITEK-2 (AST-GN67), showed that ceftriaxone, ceftazidime, and meropenem were resistant, while ciprofloxacin, piperacillin/tazobactam, and trimethoprim/sulfamethoxazole were susceptible. The minimum inhibitory concentrations (MICs) were as follows: ceftriaxone >64 μg/mL (resistant), ceftazidime >32 μg/mL, meropenem >16 μg/mL, ciprofloxacin ≤0.5 μg/mL, piperacillin/tazobactam ≤4/4 μg/mL, and trimethoprim/sulfamethoxazole ≤2/38 μg/mL.

Treatment and clinical course

Upon admission, empiric therapy with intravenous meropenem (1 g every eight hours, dose adjusted for renal function) was initiated. However, there was no clinical improvement, with persistence of fever and elevated CRP (90.2 mg/L) and procalcitonin (7.1 μg/L) on day 3 of admission. Based on antimicrobial susceptibility testing available on day 4, therapy was modified to intravenous ciprofloxacin (400 mg every 12 hours, renally adjusted). By day 7, the patient showed significant clinical improvement, as fever subsided, CRP (32.4 mg/L) and procalcitonin (1.2 μg/L) decreased, and repeat blood cultures yielded no growth. On day 8, the IJV dialysis catheter was removed due to suspected catheter-related bloodstream infection (CRBSI), and a new catheter was placed. Neurosurgical evaluation of the VP shunt revealed no clinical or laboratory evidence of shunt infection (absence of erythema, tenderness, or cerebrospinal fluid abnormalities); hence, the shunt was retained.

Supportive care included transfusion of two units of packed red blood cells for anemia and initiation of nutritional optimization. Hemodialysis was continued on a thrice-weekly schedule. At the two-week follow-up, the patient remained afebrile, with stable renal function on dialysis, no recurrence of bacteremia, and normalization of CRP (4.8 mg/L) and procalcitonin (<0.5 μg/L). Both urine and blood cultures during this period yielded no growth. Nevertheless, the subsequent course was complicated by recurrent bloodstream infections. Follow-up blood cultures isolated *Candida tropicalis*, for which antifungal therapy was commenced. Later, cultures grew extended-spectrum β-lactamase (ESBL)-producing *Escherichia coli* and *Staphylococcus epidermidis*. Subsequent blood cultures again yielded growth of Gram-negative bacteria identified as *P. apista*. Despite broad-spectrum antimicrobial therapy, antifungal treatment, and intensive supportive measures, the patient’s condition progressively deteriorated. After a few days, the patient ultimately developed refractory septic shock and died.

## Discussion

*P. apista* is an aerobic, Gram-negative, rod-shaped, non-spore-forming organism, equipped with polar flagella, a characteristic consistent with its original description by Coenye et al. (2000) [6]. These organisms grow optimally at 30-37°C and are increasingly recognized as emerging MDR pathogens. The present case adds to the expanding evidence that *Pandoraea* species, although initially identified primarily as respiratory pathogens in patients with CF, have broader clinical relevance, extending to non-CF and immunocompromised populations.

Historically, *P. apista* and related species were most often isolated from CF airways, where they are associated with chronic colonization, accelerated pulmonary decline, and difficulty in eradication. However, recent literature - including outbreaks in non-CF intensive care settings - demonstrates an evolving epidemiologic profile, in which systemic and device-associated infections are increasingly reported. Our findings align with this emerging trend, showing that *P. apista* can cause severe, disseminated infection in a non-CF host. The patient’s comorbidities - including metastatic malignancy and CKD - likely heightened susceptibility, a pattern also observed in non-CF bloodstream or device-related infections reported in the literature [5].

The isolation of *P. apista* from multiple blood and urine cultures suggests hematogenous dissemination, with a probable urinary origin secondary to obstructive uropathy. This is compatible with previously documented cases, where *Pandoraea* species demonstrated the capacity to colonize or infect non-respiratory sites, including blood, urine, and medical devices. Additionally, the possibility of VP shunt colonization is supported by earlier reports of *Pandoraea* species exhibiting strong biofilm-forming ability, contributing to persistent device-associated infections. Such behavior is consistent with the broader characteristics of the genus described in foundational taxonomic studies.

A critical aspect of this case was the organism’s MDR profile. Resistance to carbapenems - particularly meropenem - has been frequently documented for *Pandoraea* species, due to intrinsic and acquired β-lactamase mechanisms [6,7]. The resistance pattern observed here mirrors findings from prior clinical studies, in which *P. apista* demonstrated inconsistent susceptibility to carbapenems and cephalosporins, complicating empirical treatment selection. The patient’s failure to improve on meropenem and subsequent clinical response to a target-directed, susceptible agent emphasize the species’ variable and often unpredictable antimicrobial profile.

Comparative literature further supports the necessity of precise microbiological identification. Early misidentification of *Pandoraea* as *Burkholderia* spp. or other related non-fermenting Gram-negative bacilli has been widely reported [7]. Advanced sequencing technologies - including 16S rRNA sequencing and, more recently, shotgun metagenomics - have significantly improved diagnostic accuracy [8,9]. Such methods are increasingly essential, because routine biochemical assays show limited discriminatory power among phenotypically similar genera. In our case, accurate identification allowed appropriate, susceptibility-guided therapy, consistent with recent recommendations emphasizing molecular diagnostics for rare or resistant pathogens.

Recent discoveries of additional *Pandoraea* species, such as *P. fibrosis* [10], further underscore the genus’s expanding diversity and pathogenic potential. These findings collectively support the assertion that *Pandoraea* species represent emerging, clinically significant pathogens, whose epidemiology is still evolving. The limited data available regarding optimal treatment - especially in non-CF patients - highlight the importance of individualized management strategies guided by detailed susceptibility profiles.

In summary, this case illustrates the capacity of *P. apista* to cause serious, MDR infections outside the traditional CF context, particularly in immunocompromised individuals or those with indwelling devices. The findings are consistent with, yet extend beyond, existing literature documenting the organism’s virulence, diagnostic challenges, and antimicrobial resistance patterns [10]. Increased clinical awareness, improved microbiological diagnostics, and tailored antimicrobial regimens are essential for optimizing outcomes in patients affected by these rare but formidable pathogens.

## Conclusions

This case describes the first known *P. apista* CRBSI in a non-CF patient with post-revision VP shunt hydrocephalus in the Middle East. It underscores the organism’s potential as an MDR, opportunistic pathogen in severely ill, immunocompromised patients. Prompt identification and targeted susceptibility testing were essential for management, highlighting the need for clinical vigilance when rare, resistant pathogens are suspected.
